# Assessment of Phytochemical Composition and Antifungal Activity of Micropropagated *Drymis winteri* Plants

**DOI:** 10.3390/plants14203215

**Published:** 2025-10-20

**Authors:** Julia Rubio, Christian Robles-Kelly, Evelyn Silva-Moreno, Héctor Carrasco, Andrés F. Olea

**Affiliations:** 1 Instituto Ciencias Biomédicas, Universidad Autónoma de Chile, Av. del Valle Sur 534, Santiago 8580640, Chile; 2 Facultad de Medicina, Universidad Autónoma de Chile, El Llano, Santiago 8910339, Chile; christian.robles@uautonoma.cl; 3 Centro de Investigación e Innovación en Cáncer, Fundación Arturo López Pérez, Jose Manuel Infante 805, Santiago 7500921, Chile; evelyn.silva@falp.org; 4 Grupo QBAB, Instituto de Ciencias Aplicadas, Facultad de Ingeniería, Universidad Autónoma de Chile, Av. del Valle Sur 534, Santiago 8580640, Chile; hector.carrasco@uautonoma.cl

**Keywords:** *Drimys winteri*, micropropagation, callus induction, plant secondary metabolites, antifungal activity, sustainable agriculture, *Botrytis cinerea*, polygodial, drimenol

## Abstract

The search for sustainable alternatives to synthetic agrochemicals has fueled a growing interest in plant-derived bioactive compounds. *Drimys winteri* (canelo), a native Chilean tree of significant ethnobotanical importance, is a promising source of antifungal sesquiterpenes, such as polygodial and drimenol. This study describes the development of an in vitro clonal micropropagation platform for *D. winteri* that enables the production of plant material under controlled laboratory conditions, which is subsequently submitted to extraction to obtain these bioactive compounds. Four tailored culture media have been formulated for successful propagation, rooting of plantlets, and callus induction. Histological analysis confirmed the presence of meristemoids in the dedifferentiated calli. Furthermore, HPLC and GC-MS analyses indicate that phytochemical composition of extracts of *in vitro*-propagated *D. winteri* and those from mature, wild-grown trees is quite similar. This result is in line with the antifungal activity against *Botrytis cinerea* exhibited by these extracts; namely, both are comparable. This biotechnological approach offers a scalable method for producing plant-based antifungal agents, contributing to sustainable agriculture and the valorization of native genetic resources.

## 1. Introduction

In recent decades, much work has been dedicated to the search for natural products that can be used as pharmaceuticals or crop protection agents [1,2,3,4]. The study of bioactive secondary metabolites has expanded within the agricultural sciences, driven by the goal of developing plant-based products to replace synthetic chemicals like fungicides, insecticides, and pesticides. It has been shown that extensive application of synthetic crop protection agents may induce irreversible damage to ecosystems. Most of the new active molecules correspond to secondary metabolites obtained from plants [5,6]. Thus, the quest for environmentally friendly alternatives has focused on the extraction of bioactive molecules from an increasing number of plants. Generally, plants produce secondary metabolites as a response to abiotic and biotic stresses [7,8], and therefore, their phytochemical profiles depend on environmental and climate conditions [9,10,11]. Consequently, endemic plants are expected to produce different secondary metabolites. In this context, a variety of active molecules have been obtained from plants that are endemic to different regions of Chile [12,13,14]. Between them, it is worth mentioning *Drimys winteri* J.R. Forst. & G. Forst (Canelo), a native species distributed from the IV to the XII regions of Chile. This is a sacred tree with profound social and medicinal importance for the Mapuche people, one of Chile’s indigenous peoples. Its leaves and bark are used in infusions to treat various ailments, owing to their analgesic, anti-inflammatory, antifungal, and antibacterial properties, among others [14,15,16]. Phytochemical studies have confirmed the presence of primary drimane-type sesquiterpenes in leaf and bark extracts [17,18,19,20]. From these, polygodial (PubChem CID: 72503) and drimenol (PubChem CID: 3080551) inhibit spore germination and mycelial development of *Botrytis cinerea*, a phytopathogenic fungus that affects a wide range of commercially important crops [17,18]. In addition, it has been shown that the mean concentrations of both compounds measured in leaves vary in plants from different populations [20].

Preliminary studies have shown that semi-polar extracts from bark of this tree possess potent antifungal properties, paving the way for the valorization of this genetic resource and the development of commercially viable products for the agricultural sector.

However, development of alternative crop protection agents based on bark collected from mature canelo trees in their natural habitats presents limitations for potential scalable commercial production. For this reason, it is necessary to count on a reliable source of plant material with phytochemical and biological properties like those found in canelo extracts. Thus, herein we report the implementation of an in vitro clonal micropropagation platform for *D. winteri*. This approach allows extraction of target compounds from plantlets grown under controlled conditions, offering a promising and sustainable alternative [21]. The success of this technique hinges on its ability to produce large quantities of plant material in a shorter timeframe compared to traditional propagation methods, thereby increasing the efficiency of vegetative cutting production [22].

Previous in vitro studies on *D. winteri* have reported the induction of organogenic callus formation from internodal segments, apical buds, and leaves [23,24]. However, to date, there are no reports on micropropagation of *D. winteri* aimed at the extraction of bioactive components or at mass seedling production for forestry, medicinal, or ornamental purposes.

In this study, the formulation of culture media required to successfully implement an in vitro micropropagation platform for canelo is described. Results from a phytochemical study indicate that extracts obtained from in vitro-generated plants exhibit similar phytochemical profiles to those obtained from the bark of mature trees [17,18,19]. Furthermore, the observed antifungal activity of these extracts against *B. cinerea* mirrors that previously reported by our research group for extracts of mature plants. Therefore, this micropropagation platform represents a scalable biotechnological tool to produce plant-based antifungal agents.

## 2. Results

### 2.1. Propagation of Plantlets and Callus Induction in D. winteri

**Figure 1:**
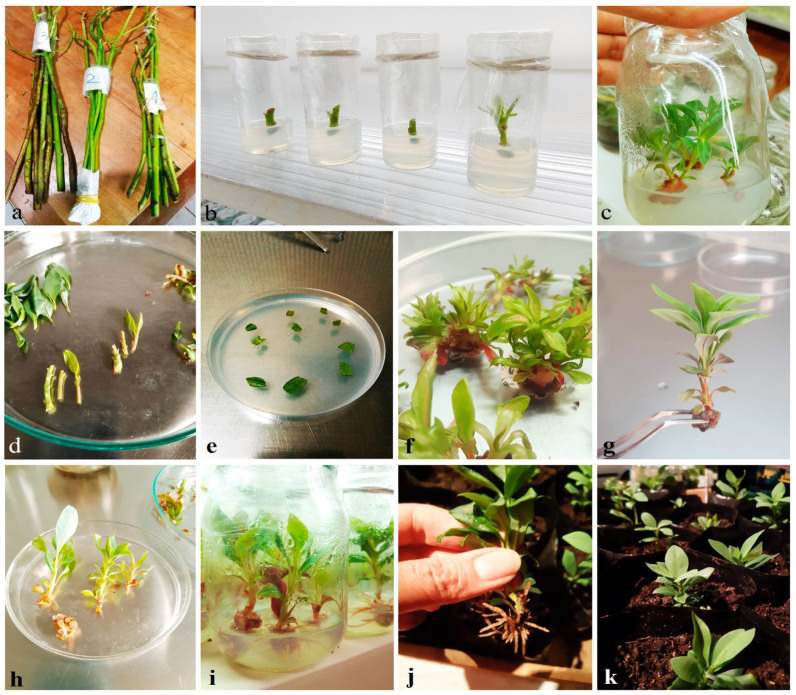
Micropropagation platform for native tree species *D. winteri* (canelo). Cuttings used for obtaining initial plant material (**a**) and shoots are established on semi-solid media (**b**). After 3 months on media culture (**c**), the obtained plant material (**d**) is used in callusing media (**e**), multiplication media (**f**), or elongation media (**g**). Shoots in medium E reach a size of 2 to 3 cm (**h**); then basal calluses are removed and established in rooting media (**i**). Plantlets with in vitro-induced roots (**j**) are established in a substrate formed by leaf soil: peat: perlite (**k**).

The in vitro propagation of canelo has been successfully achieved through four sequential stages, namely introduction (I), propagation (P), elongation (E), and rooting (R). Each specific developmental stage is well described in Figure 1.

Cuttings (a) are first established in an introduction medium (I), and the resulting shoots are cultivated in a propagation medium (P) for 45 days (b). During this phase, basal calluses are formed, which are removed at each subculture.

Lateral shoots are then isolated and transferred to an elongation medium (E) for 60 days (c). In this medium, the shoot buds develop leaves, exhibit stem thickening, and elongate to 2–3 cm (d). Selected leaf explants are placed on an organogenesis-inducing medium (e,f). Subsequently, the newly formed organogenic plantlets are isolated and transferred to an E medium (g). Finally, individual plantlets are transferred to a rooting medium (R) for 90 days (h), where they successfully form roots (i–j), enabling their acclimatization and establishment under greenhouse conditions (k). The composition of media used in these stages is detailed in Table 1.

### 2.2. Callus Induction

The callus induction process has been carried out with leaf explants (L), lateral shoots (S), and internodal segments (ISs). The media used in these experiments are described in Table 2.

Dedifferentiated calli are generated from leaf explants after 30-day incubation in darkness followed by a 30-day light/dark cycle. These calli are subcultured every 30 days on the same medium and maintained for 9 months. Callus induction from internodal segments is unsuccessful under the tested conditions.

In contrast, lateral shoots cultured in III media for 60 days generate organogenic green calli, which are subsequently transferred to elongation and rooting media according to established protocols.

### 2.3. Calli Histological Analysis

**Figure 2:**
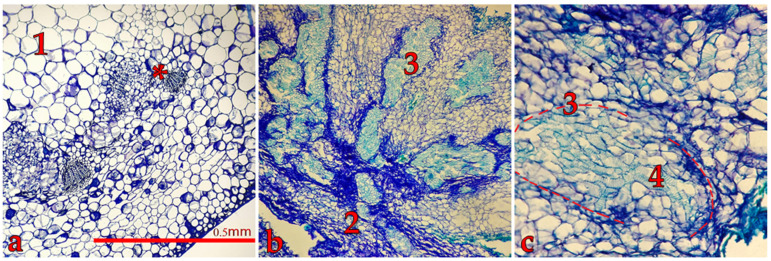
Histology of *D. winteri* stem (**a**), 100×) and callus generated under in vitro conditions (**b**) 100×; (**c**), 400×). Metachromatic toluidine blue allows us to distinguish primary cell walls (light blue) from secondary ones (dark blue). Dicotyledonous distribution (**a**): 1, pith; *, vascular vessels. Longitudinal slice of de-differentiation zone (**b**): 2, parenchyma, epidermis, cuticle; 3, meristemoids. Transversal meristemoid growth zone (**c**): 3, meristemoids; 4, dashed red line limiting meristemoid area.

Histological analysis of callus tissue reveals rounded structures known as meristemoids (Figure 2b,c).

These structures comprise groups of mature cells that, under hormonal stimuli, dedifferentiate into isodiametric, thin-walled cells, characteristic of meristems. In *D. winteri*, metachromatic staining with toluidine blue allows us to distinguish between primary (light blue) and secondary (dark blue) cell walls, highlighting the actively dividing zones.

### 2.4. Analysis of In Vitro Plant and Callus Crude Extracts

#### 2.4.1. Phytochemical Analysis

**Figure 3:**
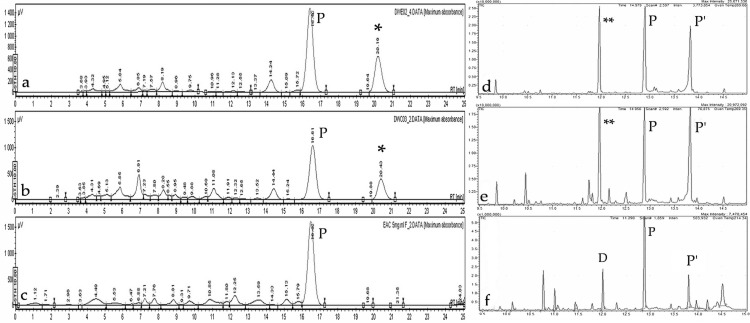
Phytochemical profiles obtained for DWE02 (in vitro plant) (**a**,**d**), DWC03 (basal callus) (**b**,**e**), and EAC (mature bark) (**c**,**f**) extracts. (**a**–**c**) HPLC, using acetonitrile/water 70:30 as mobile phase: P, polygodial; * unidentified compound. (**d**–**f**) GC-MS: P, polygodial; D: drimenol; P’ different form of polygodial; ** compound similar to drimenol.

The chemical composition of ethyl acetate extracts obtained from *D. winteri* bark (EAC, collected from Chiloé Island, Chile), in vitro-propagated *D. winteri* plants (DWE02), and callus cultures (DWC03) was assessed by high-performance liquid chromatography (HPLC) and gas chromatography–mass spectrometry (GC-MS), using polygodial, drimenol, and isodrimenin as reference standards. Typical HPLC and GC chromatograms are shown in Figure 3.

A peak with a retention time (RT) coinciding with the polygodial standard is observed across all HPLC chromatograms, confirming that this compound is produced by in vitro-derived tissues (HPLCs of standards are shown in Figures S1 and S2, Supplementary Material). The relative abundance, obtained from comparison of peaks area, is higher in DWE02 than in DWC03. On the other hand, drimenol and isodrimenin peaks are not detected in either extract (HPLCs of standards and that obtained for EAC are shown in Figure S1, Supplementary Material). Interestingly, an additional peak appears at a later RT in both DWE02 and DWC03 that is not observed in the EAC extract. Probably, this peak corresponds with a novel drimane-type derivative synthesized under clonal micropropagation culture conditions, as previously reported for tissue culture-derived metabolites [7,8].

Gas chromatography–mass spectrometry (GC-MS) allows a more precise characterization of extracts’ chemical profiles (Figure 3d–f). The MS detector provides a fragmentation pattern for each peak observed in the GC chromatogram. Thus, drimenol appears with a RT of 12.015 min, and gives a molecular ion at *m*/*z* 222 (Figure S3, Supplementary Material). On the other hand, two forms of polygodial are observed at RT of 12.902 and 13.819 min, with a molecular ion at *m*/*z* 234 and diagnostic fragments at *m*/*z* 206, 121, 110, 109, and 91 (Figure S4, Supplementary Material). Both metabolites have been identified by comparison of the obtained fragmentation pattern (Figure 3d–f, Table S1, Supplementary Material) with data found in the NIST Mass Spectral Library (Scientific Instrument Services). Interestingly, chromatograms of the DWC03 extract show a peak at a RT of 9.590 min, characterized by fragments overlapping with polygodial (*m*/*z* 206, 121, 110, 109, and 91), but there is no sign of the molecular ion, suggesting that this signal corresponds to a compound with partial similarity to polygodial.

Extracts obtained from calli, induced under varying hormonal conditions, exhibit distinct chemical compositions. Notably, calli derived from IV and V treatments, which employ picloram (4-Amino-3,5,6-trichloro-2-pyridinecarboxylic acid) as the main growth regulator (Table 2), produce extracts with no detectable polygodial. In contrast, calli obtained from leaves or basal calli, and formed through direct stem contact with the semi-solid medium, lead to profiles like those shown by extracts from in vitro plants and mature tree bark, including peaks consistent with drimenol and polygodial (Figure 3a–c).

These results show that phytochemical profiles of EAC, DWE02, and DWC03, obtained by HPLC, seem to be relatively similar, whereas chemical differences, especially regarding drimane-type sesquiterpenes, are observed in GC-MS.

Due to the high-water content of calli, extraction yields were lower compared to other tissues. Therefore, basal calli—representing consistent chemical profiles with in vitro plants and mature bark—were selected for downstream biological activity assays.

#### 2.4.2. Antifungal Activity of Crude Extracts

**Figure 4:**
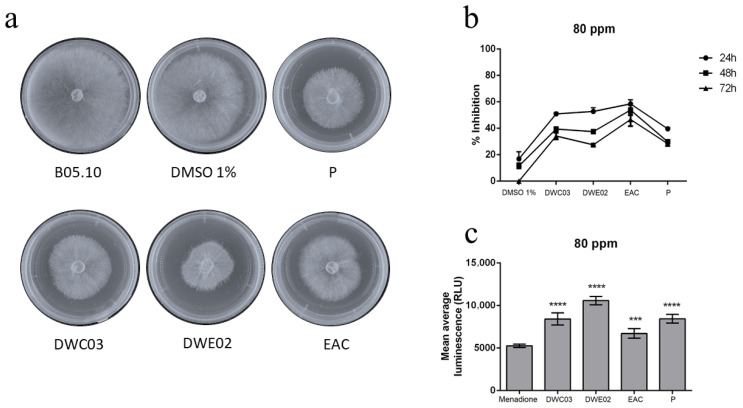
Antifungal effects on *B. cinerea* B05.10. (**a**) Radial growth inhibition. (**b**) Mycelial growth inhibition percentages. (**c**) ROS production. Mean ± SD, n = 9 per group. * *p* < 0.05, ** *p* < 0.01, *** *p* < 0.001, **** *p* < 0.0001 vs. DMSO or menadione control.

The effect of crude extracts on mycelial growth inhibition of *B. cinerea* B05.10 has been assessed by radial growth inhibition assays. *B. cinerea* was exposed to different treatments, DWC03, DWE02, EAC, and polygodial as positive control, at two concentrations 80 ppm and 160 ppm. All treatments significantly reduced fungal growth as compared to the negative control (DMSO), and no significant differences were found for both concentrations. These findings are in line with previous reports of polygodial inhibiting *B. cinerea* growth by up to 50% at similar concentrations [17,25]. In Figure 4, the results are shown, obtained at 80 ppm.

Reactive oxygen species (ROS) production was evaluated after 2 h of exposure to each extract, with luminescence measured in the presence of menadione as a ROS-inducing control. All treatments triggered significant H_2_O_2_ generation, suggesting oxidative stress (Figure 4c).

### 2.5. Gene Expression Analysis in B. cinerea

**Figure 5:**
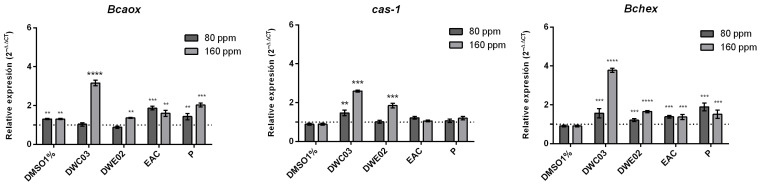
Gene expression in *B. cinerea* after 24 h exposure. Relative expression levels of Bcaox, Bchex, and cas-1 at 80 and 160 ppm. Mean ± SD, n = 9 per group. * *p* < 0.05; ** *p* < 0.01; *** *p* < 0.001; **** *p* < 0.0001. The dotted line indicates the normalized expression level (=1), corresponding to the calibrator/control in the 2^−ΔΔCT^ method (Ref. [16]). Values below 1 represent downregulation, whereas values above 1 denote upregulation.

Gene expression changes were assessed in *B. cinerea* spores exposed for 24 h to different treatments (DWC03, DWE02, and EAC) and polygodial. Three genes were analyzed: Bcaox, involved in oxidative stress response [26]; Bchex, encoding the major protein of the Woronin body, associated with hyphal damage [27]; and cas-1, an ortholog of casA, which encodes a metacaspase related to fungal programmed cell death (PCD) [28]. Two concentrations were used (80 and 160 ppm) to determine whether the transcriptional responses were dose-dependent. Results are shown in Figure 5.

Expression of Bcaox is significantly upregulated by polygodial and EAC (bark extract) at both tested concentrations, whereas extracts DWCO3 (basal callus) and DWE02 (in vitro plant) are active only at 160 ppm. This effect can be attributed to mitochondrial dysfunction, likely caused by damage to the ATP-synthesizing complexes located in the inner membrane. The differences in activity exhibited by the extracts are possibly due to higher purity or presence of additional drimane-type sesquiterpenes in mature plant extracts.

For Bchex, a four-fold increase was observed in response to DWC03 at 160 ppm, while DWE02, EAC, and polygodial produced a maximum two-fold increase at the same concentration. At 80 ppm, the effects were notably reduced. The overexpression of Bchex may reflect cellular responses to damage at the germ tube level, consistent with inhibited conidial germination.

Interestingly, cas-1 expression was not induced either by polygodial or EAC, but treatments with DWC03 and DWE02 at 160 ppm led to a significant three-fold upregulation. Interestingly, polygodial showed no activity, and, therefore, these results suggest that activation of fungal PCD pathways can be attributed to the other components present in callus and in vitro plant extracts.

Plant defense molecules are known to induce PCD in *B. cinerea* by activating ROS or directly targeting the apoptosis machinery [29,30]. Studies in *Aspergillus nidulans* have shown that casA overexpression inhibits hyphal growth and leads to morphological changes consistent with apoptosis [31,32]. Although ROS induction likely contributes to this response, it is important to note that apoptosis can also be triggered independently of H_2_O_2_ activity. Previous short-term (6 h) qPCR assays from our group did not detect cas-1 overexpression, supporting the hypothesis that extended exposure to bioactive compounds is required to trigger apoptosis-related gene expression in *B. cinerea*.

## 3. Discussion

The micropropagation platform developed for *D. winteri* enabled the successful generation of complete plantlets from cuttings, demonstrating that the hormonal formulations applied at each stage (introduction, propagation, elongation, and rooting) effectively supported proper morphophysiological development. The recurrent formation of basal calluses during the propagation stage may be associated with the presence of auxins in the medium, suggesting a hormonal balance conducive to partial dedifferentiation. This phenomenon was later leveraged for the induction of either dedifferentiated or organogenic calli, depending on the explant type and the hormonal combination used.

Callus induction from leaf explants proved significantly more efficient than from internodal segments. In some cases, green organogenic calli were generated, highlighting the species’ differential response capacity to hormonal stimuli. The presence of meristemoid structures observed in histological analyses supports this plasticity, as these cell aggregates are characteristic of tissues with regenerative potential.

Phytochemical analyses performed by HPLC and GC-MS provide complementary insights into the drimanoid profile of *D. winteri* bark and in vitro-derived materials. Mature tree bark from Chiloé island (EAC) consistently displayed polygodial as the dominant metabolite, in agreement with previous phytochemical reports [19,20,25]. On the other hand, in vitro plants (DWE02) largely reproduced this bark profile, showing a strong polygodial signal in HPLC. However, under GC–MS conditions, polygodial could not be detected, most likely due to its thermal lability and the extensive fragmentation caused by electron impact ionization [33]. Sesquiterpene dialdehydes, such as polygodial, are particularly unstable under these conditions, and matrix effects inherent to in vitro extracts may further suppress detection [34]. Thus, the discrepancy between HPLC and GC–MS does not necessarily reflect a true absence of polygodial in DWE02, but rather the limitations of GC–MS for this compound. By contrast, callus extracts (DWC03) show lower levels of polygodial and distinct peaks not present in bark or in vitro plants. HPLC revealed a unique metabolite at ~16.8 min, whereas GC–MS detected a peak with mass fragments overlapping with those of polygodial, but its molecular ion was lacking. These features suggest accumulation of structurally related drimanoid derivatives, possibly isodrimenin or novel lactones [35,36]. Such metabolic divergence indicates that callus cultures may activate alternative biosynthetic pathways, leading to chemical diversity beyond that observed in bark or whole-plant extracts.

Regarding antifungal activity, all tested extracts inhibited the growth of *B. cinerea*, confirming their antifungal potential. Membrane integrity and reactive oxygen species (ROS) assays showed effects comparable to those of the positive control (70% ethanol), indicating that the extracts compromise fungal cell membranes and induce oxidative stress.

Gene expression analysis in *B. cinerea* exposed to the extracts provided additional insight into the mechanisms of toxicity. Upregulation of Bcaox suggests mitochondrial dysfunction associated with oxidative stress, while Bchex induction reflects hyphal damage. The specific induction of cas-1 by basal callus and in vitro plant extracts—but not by mature bark or pure polygodial—indicates that certain components exclusive to in vitro cultures may activate programmed cell death (PCD) pathways distinct from those triggered by polygodial.

Altogether, the results demonstrate that in vitro-derived tissues not only enable clonal propagation of a valuable native species but also represent a promising source of bioactive compounds with antifungal properties. The use of plant cell cultures as platforms to produce secondary metabolites—especially those undetectable in mature tissues—opens new possibilities for the biotechnological valorization of *D. winteri*.

## 4. Materials and Methods

### 4.1. Plant Material and Micropropagation

Green stems were collected from three different locations near Cauquenes, in the Maule Region of Chile, during the early summer of 2018. The sterilization procedure is as follows: (1) branches are washed with running tap water and commercial soap for 10 min; (2) rinsed with sterile distilled water; (3) immersed for 1 h in a solution of Benlate^®^ and Captan^®^ prepared according to the manufacturer’s instructions; (4) immersed for 20 min in a 10% sodium hypochlorite solution containing three drops of Tween 20 per 100 mL; and (5) rinsed three times with sterile distilled water.

The sterilized branches were cut into 1 cm segments, each containing at least one dormant bud. These cuttings were placed in flasks containing an introduction medium (I) that did not contain antibiotics or antifungal agents. After 2–4 weeks, the buds sprouted, producing plantlets with 3 to 5 leaves. This material served as the source for subsequent callus induction and micropropagation.

For micropropagation, various combinations of hormone concentrations were tested, modifying previously published media [23,24]. The material was cultured in glass flasks containing 30 mL of semi-solid WPM (Woody Plant Medium, Caisson) [37], supplemented with 30 g/L sucrose and various concentrations of phytohormones: 1-naphthaleneacetic acid (NAA) (0.6, 0.8, and 1.0 mg/L), 6-benzylaminopurine (BAP), 6-furfurylaminopurine (KIN), gibberellic acid (GA3) (0.01 and 0.05 mg/mL), and adenine sulfate (20 and 40 mg/L). The pH of the medium was adjusted to 5.5 and solidified with 7 g/L plant agar. Cultures were maintained at 24 ± 2 °C with 40–60% relative humidity, under illumination from daylight fluorescent lamps (48 μmol m^−2^ s^−1^) with a 16/8 h (light/dark) photoperiod.

### 4.2. Callus Induction and Morpho-Histological Characterization

Callus induction is performed using explants from the in vitro-propagated plants, including leaf explants (L), internodal segments (ISs), and shoot explants (S). These explants are randomly placed in Petri dishes containing 15 mL of either semi-solid MS medium [38] or WPM. A range of phytohormones at different concentrations (0.1 and 1.0 mg/L of NAA, BAP, KIN, PIC, thidiazuron (TDZ), and zeatin (Z)), along with sucrose (10% and 20%), were tested. The pH is adjusted to 5.5 and the medium was solidified with 7 g/L plant agar. The cultures are incubated for 30 days in total darkness, followed by a 16/8 h (light/dark) photoperiod with illumination at 48 μmol/m^2^s. The optimal treatment was determined based on the highest Callus Induction Frequency (CIF) [39] and other variables such as callus induction time, morphology (friable, compact, oxidized), and the presence or absence of organogenesis [32]. All experiments were repeated twice with 20 replicates per treatment.

For histological examination, friable callus tissue is fixed in FAA (formalin:acetic acid:ethanol, 5:5:90 *v*/*v*/*v*) for 24 h, stored in 70% ethanol, dehydrated in a graded ethanol series, and embedded in paraffin. Sections of 8–10 μm are cut using a microtome (Microm HM 325, Thermo Scientific, Walldorf, Germany) and stained with Safranin-O according to reported protocols [40,41].

### 4.3. Extraction and Analytical Assays

Crude extracts from *D. winteri* in vitro-induced callus (from leaf sections), basal callus, whole in vitro-micropropagated plants, and bark from mature trees (collected in Chiloé) have been prepared following established protocols [42,43]. Between 10 and 30 g (wet weight) of callus or whole in vitro plantlets (without roots or basal callus) are dried at room temperature for one week, macerated in ethyl acetate (Merck Millipore, Darmstadt, Germany), and incubated at 25 ± 2 °C for 48 h. The suspensions are filtered and concentrated using a rotary evaporator. The resulting extracts are dissolved in acetonitrile (1 mg/mL) for HPLC analysis and in DMSO (final concentration of 1%) for biological activity assays.

Phytochemical composition is analyzed by HPLC (JASCO, Tokyo, Japan) in an instrument equipped with a quaternary pump (PU-2089, JASCO) and a diode array detector (MD-2010, JASCO) scanning from 195 to 650 nm. Briefly, a 20 μL aliquot of the semipolar extract is injected into a 100-5-C18 column 4.6 mm × 150 mm (Kromasill, Nouryon, Sweden). Mixtures of acetonitrile:water at ratios of 60:40, 70:30, and 80:20, are used as mobile phase, with a flow rate of 0.5 mL/min. A polygodial sample is used as the standard for calibration. Gas chromatography–mass spectrometry (GC–MS) analyses are performed using a single-quadrupole GC (QP2010 SHIMADZU, Tokyo, Japan) equipped with a Rtx-5MS capillary column (30 m length; 0.25 mm diameter; 0.25 μm thickness) (Restek, Bellofonte, PA, USA), coupled to a quadrupole mass-selective detector and three ionization sources: EI, positive chemical ionization (PCI), and negative chemical ionization A DB-17MS. The injector temperature is set to 250 °C, and helium is used as carrier gas at a constant flow rate of 1.61 mL min^−1^. The oven temperature program is started at 60 °C, held for 1 min, followed by a ramp of 15 °C min^−1^ to 285 °C, and held for 4 min. Mass spectra are recorded under electron impact ionization (EI) at 70 eV, scanning in the range of *m*/*z* 40–500. The transfer line temperature is set to 280 °C. Injection (1 μL) is carried out in splitless mode. Chromatogram components are identified by searching in the NIST 11 library (NIST, Mass Spectral Search Program v.2.0g) and SpectraBase^®^ (Bio-Rad Lab, Hercules, CA, USA). When applicable, the literature-reported fragmentation patterns for drimanoid compounds (e.g., isodrimenin) have also been consulted to interpret unidentified drimanoid-like peaks.

### 4.4. Antifungal Properties Against Botrytis Cinerea

The biological activity of crude extracts obtained from basal calluses (DWC03), in vitro plants (DWE02), and mature tree bark (EAC) against *B. cinerea* strain B05.10 has been evaluated as previously described [17,18,44]. All treatments are dissolved in DMSO/water (1:100) and applied in the amounts needed to reach final concentrations of 80 ppm and 160 ppm. Germination percentage is determined by counting the number of germinated conidia (defined as those with a germ tube length greater than or equal to the conidial diameter). ROS production is measured by using the ROS-Glo™ H_2_O_2_ Assay kit (Promega, USA), and a luminometer Tecan Infinite M200pro (Männedorf, Switzerland) [45]. Each experiment is performed in triplicate.

Changes in gene expression have been assessed by using protocols similar to those previously described [17]. Briefly, conidia are inoculated into potato-dextrose broth (1.5 mL) containing each treatment (80 and 160 ppm), and cultured with shaking in darkness for 24 h at 21 ± 1 °C. Then conidia are washed and ground with a bead beater using silica microbeads. RNA is extracted with RA1 lysis buffer (Macherey-Nagel, Germany). At least three independent RNA preparations are analyzed. Each target gene is measured in triplicate, and the mean Cq value is calculated. Relative gene expression is determined using the 2^−ΔΔCT^ method [46], normalizing to the Ubiquitin-conjugating enzyme E2 (UbcE) gene. Statistical differences are evaluated with a one-way ANOVA, followed by Sidak’s multiple comparison test (*p* < 0.05) using GraphPad Prism 6.

## 5. Conclusions

Crude extracts obtained through a semipolar extraction method from basal calli and in vitro-propagated canelo plants exhibit antifungal activity against the phytopathogenic fungus *B. cinerea* B05.10. These activities were evaluated and compared to that exhibited by polygodial, the most abundant antifungal compound identified in mature canelo bark. Results confirm that extracts of *D. winteri* bark contain polygodial as the main drimanoid metabolite, with drimenol and isodrimenin occurring in lower amounts. In vitro-propagated plants extract (DWE02) largely replicate this phytochemical fingerprint, confirming their potential as a scalable source of polygodial. However, polygodial is not observed under the applied conditions of GC–MS analyses, highlighting the analytical challenges associated with detecting sesquiterpene dialdehydes in complex matrices. In contrast, basal callus tissue extract (DWC03) has reduced polygodial content and presence of alternative drimanoid metabolites, pointing to metabolic redirection under dedifferentiated culture conditions. This dual outcome—phytochemical fidelity in micropropagated plants and metabolic innovation in calluses—underscores the value of in vitro systems, both for conserving and expanding the chemical repertoire of *D. winteri*.

The mechanism of action of both calluses and in vitro plant extracts involves clear disruption of fungal cell membrane integrity, induction of reactive oxygen species (ROS), and overexpression of genes associated with oxidative stress and germ tube development. The comparison of these responses with those induced by polygodial alone [17,25] suggests that the extracts act during early stages of spore germination. This supports the hypothesis that the presence of polygodial in the extracts contributes to the observed inhibition of *B. cinerea* mycelial growth through effective interference with spore germination.

Moreover, based on the in vitro results, callus and in vitro plant extracts may have long-term effects on anti-apoptotic mechanisms described for *B. cinerea.* Further studies focusing on specific apoptotic markers—such as DNA fragmentation, caspase activity, and changes in the expression of anti-apoptotic genes like Bcbir1 or Bcnma—will be necessary to address this hypothesis in greater detail [29,47].

Overall, our findings suggest that, consistent with previous studies, extracts from in vitro-derived calli and plants exhibit phytochemical profiles like those of mature canelo bark collected from natural environments, which is the traditional source of polygodial. This work lays a solid foundation for future efforts aimed at scaling up the micropropagation of *D. winteri* using temporary immersion bioreactor (TIB) systems. Such a scalable platform could significantly enhance the biotechnological potential of native species, supporting sustainable crop protection and efficient plant production.

## Figures and Tables

**Table 1:** In vitro media culture used for clonal micropropagation platform of *D. winteri*.

Woody Plant Medium (WPM)		With Vitamins		Without Vitamins	
		I **^2^**	P	E	R
Hormones ^1^ (μM)	NAA	0.55	0.28	0.55	5.49
	BAP	-	3.55	3.55	-
	KIN	4.65	-	-	-
	GA3	0.15	-	-	-
Others (mg/L)	Ascorbic acid	100	50	50	-
	Citric acid	100	50	50	-
	Adenine sulfate	-	40	-	

^1^ Abbr. NAA, 1-naphthaleneacetic acid; BAP, 6-benzylaminopurine; KIN, kinetin; GA3, gibberellic acid; ^2^ modified from Jordan, Ref. [24]. In vitro media culture used for the clonal micropropagation platform are labeled as introduction (I), propagation (P), elongation (E), and rooting (R) medium.

**Table 2:** Hormonal combinations used for the induction of dedifferentiated in leaf explants (L), lateral shoots (S), and internodal segments (ISs).

Media Formulation	Friable and Compact Calluses						Leaf or Root Organorganogenesisgenesis			
	MS						WPM			
	I ^1^	II	III	IV	V	VI	VI(1)	VI(1)	VI(2)	VII
Vegetal material	L	S	L	L	L	L	S	L	IS	IS
N° of used explants	40	40	40	40	40	40	40	40	40	40
N° of generated calluses	20	6	8	32	30	12	24	16	0	0
N° of eliminated calluses	20	34	32	0	0	28	16	24	40	40
CIF (%)	50	15	20	80	75	30	60	40	0	0

^1^ modified from Ref. [23]. I: BAP/Z; II: KIN/TDZ; III: NAA/TDZ; IV: PIC/KIN; V: 3PIC; VI: KIN/NAA using half-strength MS media; VI(1): KIN/NAA using half-strength MS media; VI(2): KIN/NAA using half-strength WPM media; VII: NAA/Z. Abbreviations: BAP, 6-benzylaminopurine; KIN, kinetin; NAA, 1-naphthaleneacetic acid; PIC, Picloram; TDZ, thidiazuron; Z, zeatin.

## Data Availability

Data are contained within the article and Supplementary Materials.

## Supplementary Materials

The following supporting information can be downloaded at: https://www.mdpi.com/article/10.3390/plants14203215/s1, Figure S1: HPLC chromatograms. Mixture of acetonitrile: water at ratio of 80:2; Figure S2: HPLC chromatograms. Mixture of acetonitrile: water at ratio of 70:30; Figure S3: GC-MS chromatogram of Drimenol; Figure S4: GC-MS chromatogram of Polygodial; Table S1: Data from the GC/MS

## Abbreviations

The following abbreviations are used in this manuscript:

BAP	6-benzylaminopurine
KIN	Kinetin (6-furfurylaminopurine)
NAA	1-naphthaleneacetic acid
PIC	Picloram (4-Amino-3,5,6-trichloro-2-pyridinecarboxylic acid)
TDZ	thidiazuron
Z	zeatin
GA3	gibberellic acid
ROS	reactive oxygen species
WPM	Woody Plant Medium
MS	Murashige and Skoog Medium
DWC03	Basal callus extract
DWE02	In vitro propagated plant extract
EAC	Bark extract
