# Supplementary material for: Assessment of Phytochemical Composition and Antifungal Activity of Micropropagated Drymis winteri Plants
Source: Plants (Basel). 2025 Oct 20;14(20):3215. doi: 10.3390/plants14203215 (PMC12566860; doi:10.3390/plants14203215)
Supplement: Supplementary file 1 [file plants-14-03215-s001.zip › plants-3904851-supplementary.pdf]

plants

MDPI

Article

Assessment of Phytochemical Composition and Antifungal Activity of Micropropagated Drymis Winteri Plants

Julia Rubio [1,*], Christian Robles-Kelly [2], Evelyn Silva-Moreno [3], Héctor Carrasco [4] and Andrés F. Olea [4,*]

[1] Instituto Ciencias Biomédicas, Universidad Autónoma de Chile, Av. del Valle Sur 534, Santiago 8580640, Chile

[2] Facultad de Medicina, Universidad Autónoma de Chile, El Llano, Santiago 8910339, Chile; christian.robles@uautonoma.cl

[3] Centro de Investigación e Innovación en Cáncer, Fundación Arturo López Pérez, Jose Manuel Infante 805, Santiago 7500921, Chile; evelyn.silva@falp.org

[4] Grupo QBAB, Instituto de Ciencias Aplicadas, Facultad de Ingeniería, Universidad Autónoma de Chile, Av. del Valle Sur 534, Santiago 8580640, Chile; hector.carrasco@uautonoma.cl

* Correspondence: jrubioa@gmail.com (J.R.); andres.olea@uautonoma.cl (A.F.O.)

Academic Editor: Hazem Salaheldin Elshafie

Received: 16 September 2025

Revised: 9 October 2025

Accepted: 16 October 2025

Published: 20 October 2025

Citation: Rubio, J.; Robles-Kelly, C.; Silva-Moreno, E.; Carrasco, H.; Olea, A.F. Assessment of Phytochemical Composition and Antifungal Activity of Micropropagated Drymis Winteri Plants. *Plants* **2025**, *14*, 3215. https://doi.org/10.3390/plants14203215

Copyright: © 2025 by the authors. Licensee MDPI, Basel, Switzerland. This article is an open access article distributed under the terms and conditions of the Creative Commons Attribution (CC BY) license (https://creativecommons.org/licenses/by/4.0/).

Supplementary Materials: The following supporting information can be downloaded at: https://www.mdpi.com/article/10.3390/plants14203215/s1:

- **Figure S1**: HPLC chromatograms. Mixture of acetonitrile: water at ratio of 80:20, are used as mobile phase, with a flow rate of 0.5 mL/min, during 30 min. In the figure: a, EAV (cortex extract from Valdivia); b, EAC (cortex extract from Chiloé); c, Polygodial; d, Drimenol; e, Isodrimenin.
- **Figure S2**: HPLC chromatograms. Mixture of acetonitrile: water at ratio of 70:30, are used as mobile phase, with a flow rate of 0.5 mL/min, during 30 min. In the figure: a, EAO (cortex extract from Osorno); b, DWC01 (basal callus extract); c, Polygodial; d, Drimenol.
- **Figure S3**: GC-MS chromatogram of Drimenol
- **Figure S4**: GC-MS chromatogram of Polygodial
- **Table S1**. Data from the GC/MS

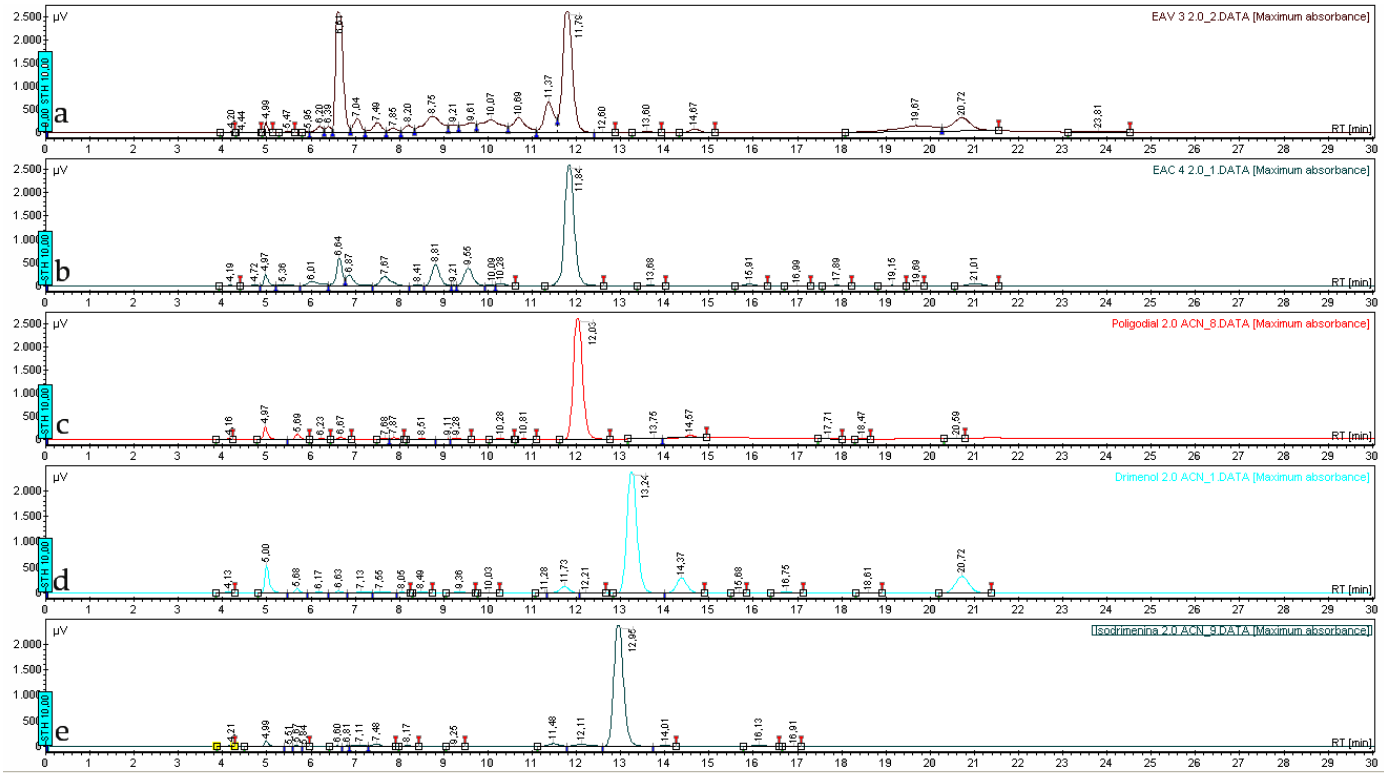

Figure S1: HPLC chromatograms. Mixture of acetonitrile: water at ratio of 80:20, used as mobile phase, with a flow rate of 0.5 mL/min, during 30 min. In the figure: a, EAV (bark extract from Valdivia); b, EAC (bark extract from Chiloé); c, Polygodial; d, Drimenol; e, Isodrimenin.

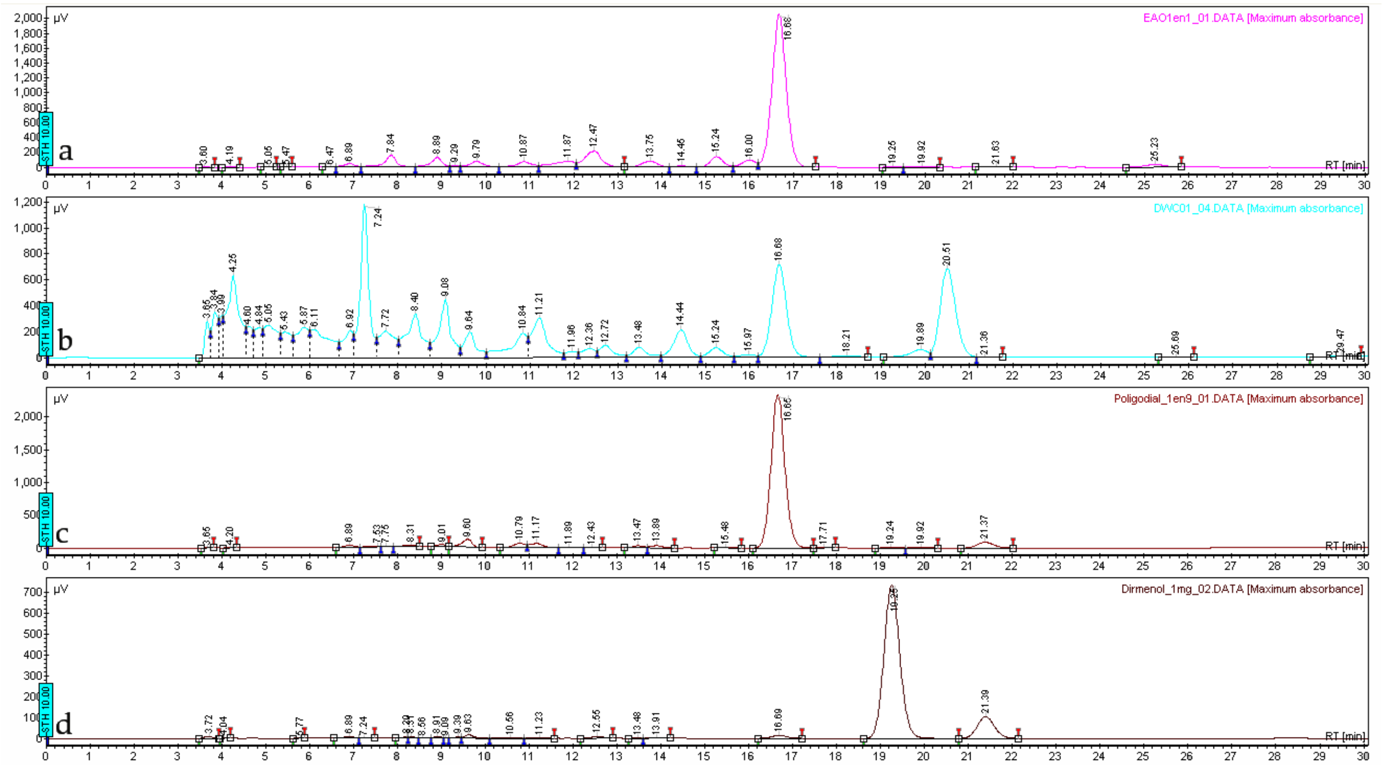

Figure S2: HPLC chromatograms. Mixture of acetonitrile: water at ratio of 70:30, used as mobile phase, with a flow rate of 0.5 mL/min, during 30 min. **a**: EAO (cortex extract from Osorno); **b**, DWC01 (basal callus extract); **c**, Polygodial; **d**, Drimenol.

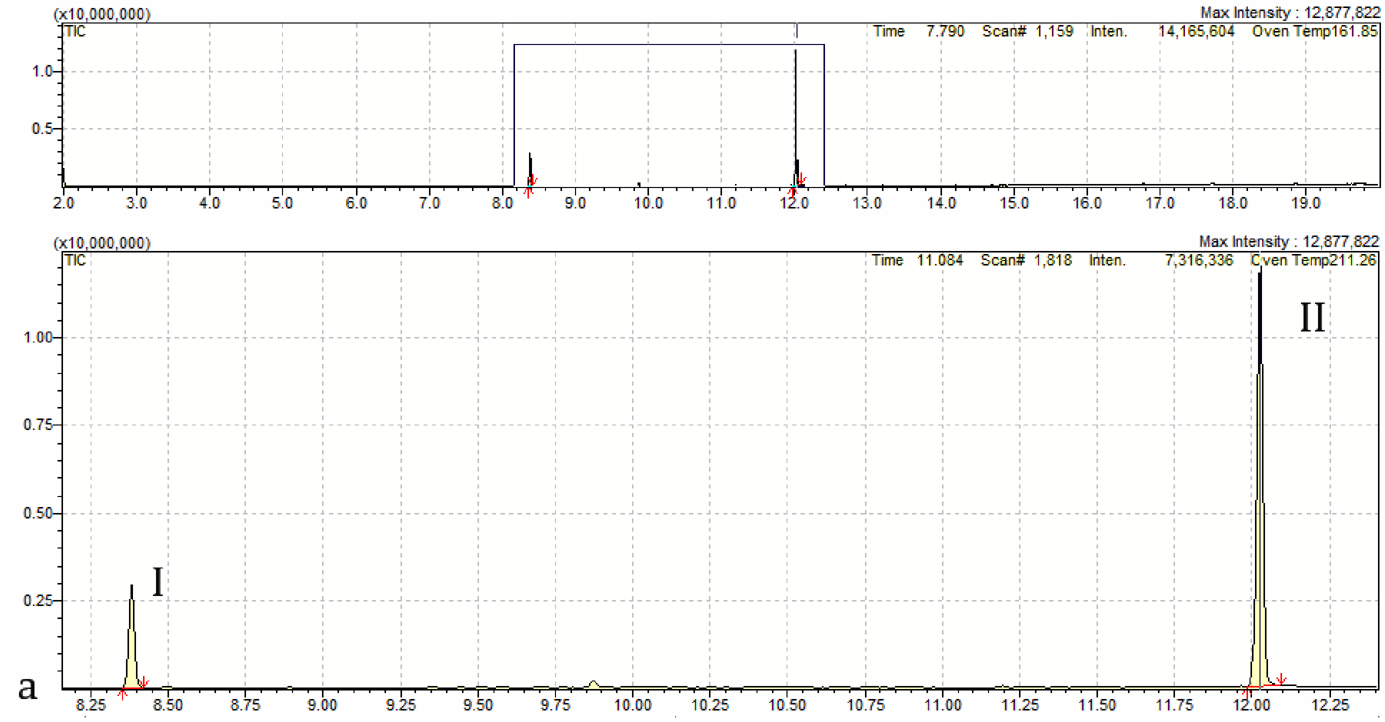

Figure S3: GC-MS chromatogram of Drimenol. Above is shown the complete chromatogram with two peaks. Below a zoom of the same chromatogram with peaks I and II. The peak at 12.05 min gives a molecular ion m/z =222, and fragments at m/z = 109, 124, 69, 81, 55

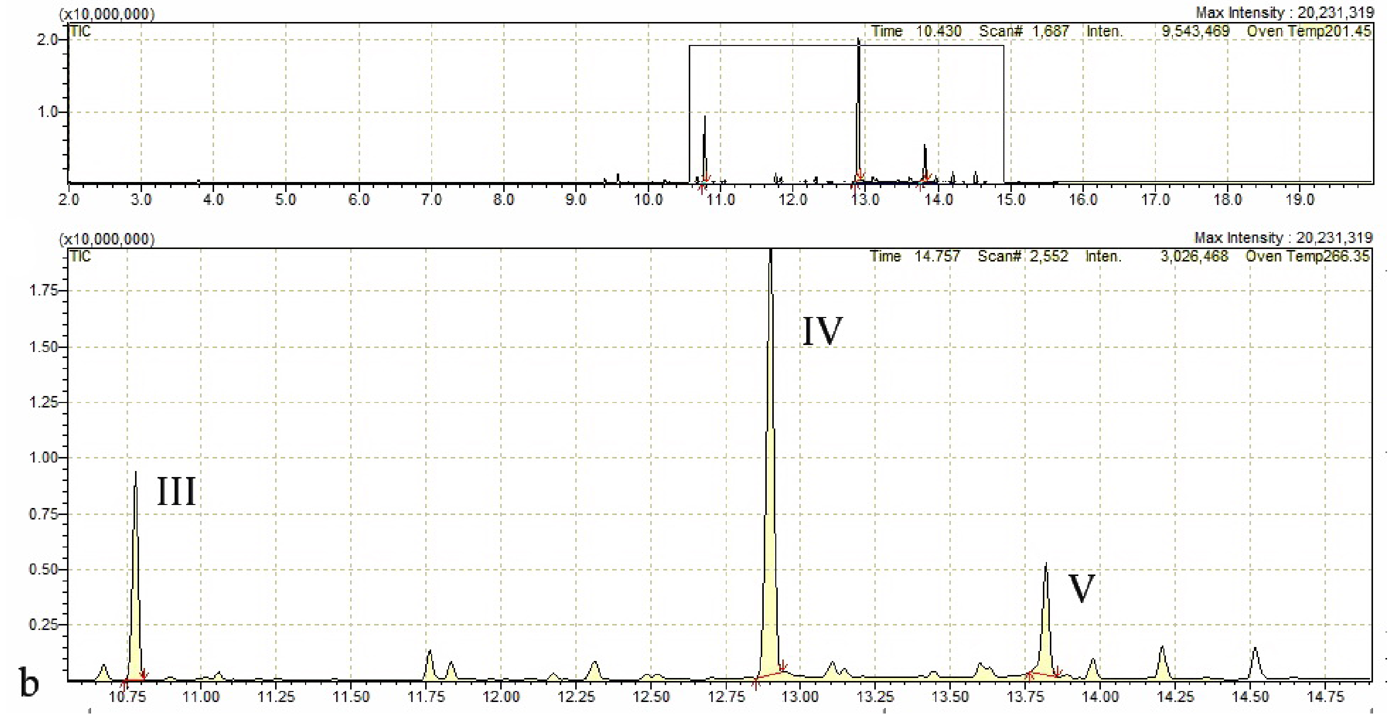

Figure S4: GC-MS chromatogram of Polygodial. Above is shown the complete chromatogram with two peaks. Below a zoom of the same chromatogram with peaks III, IV and V. The peaks at 12.9 and 13.8 min gives a molecular ion m/z = 234, and fragments at m/z = 121, 206, 110, 109, 91

Table S1. Data from the GC/MS.

Sample	RT (min)	Base Ion (m/z)	Fragments (m/z)	M^+ (m/z)	Observations
Drimenol (II)	12.015	109	124, 95, 81, 69 55	222	Laboratory purified Drimenol
Polygodial (IV)	12.902	121	206, 110, 109, 93, 91	234	Principal reference of Polygodial (laboratory purified)
Polygodial (V)	13.819	121	206, 110, 109, 91	234	Additional match of polygodial

Disclaimer/Publisher's Note: The statements, opinions and data contained in all publications are solely those of the individual author(s) and contributor(s) and not of MDPI and/or the editor(s). MDPI and/or the editor(s) disclaim responsibility for any injury to people or property resulting from any ideas, methods, instructions or products referred to in the content.
